# Drug costs in the management of metastatic castration-resistant prostate cancer in Canada

**DOI:** 10.1186/1472-6963-14-252

**Published:** 2014-06-13

**Authors:** Alice Dragomir, Daniela Dinea, Marie Vanhuyse, Fabio L Cury, Armen G Aprikian

**Affiliations:** 1 Department of Surgery, Division of Urology, McGill University, 1650 Cedar Avenue, Montreal, Quebec H3G 1A4, Canada; 2 Research Institute of McGill University Health Center, 2155 Guy St, Montreal, Quebec H3H 2R9, Canada; 3 Faculty of Pharmacy, University of Montreal, CP 6128 Succursale Centre-Ville, Montreal, Quebec H3C 3 J7, Canada; 4 McGill University Health Center, 1650 Cedar Avenue, Montreal, Quebec H3G 1A4, Canada; 5 Department of Oncology, Division of Medical Oncology, McGill University, 1650 Cedar Avenue, Montreal, Quebec H3G 1A4, Canada; 6 Department of Oncology, Division of Radiation Oncology, McGill University, 1650 Cedar Avenue, Montreal, Quebec H3G 1A4, Canada

**Keywords:** Metastatic castration-resistant prostate cancer, Treatments for advanced prostate cancer, Cost of treatments, Markov model, Cost of drugs for metastatic castration-resistant prostate cancer in Canada

## Abstract

**Background:**

For Canadian men, prostate cancer (PCa) is the most common cancer and the 3rd leading cause of cancer mortality. Men dying of PCa do so after failing castration. The management of metastatic castration-resistant prostate cancer (mCRPC) is complex and the associated drug treatments are increasingly costly. The objective of this study was to estimate the cost of drug treatments over the mCRPC period, in the context of the latest evidence-based approaches.

**Methods:**

Two Markov models with Monte-Carlo microsimulations were developed in order to simulate the management of the disease and to estimate the cost of drug treatments in mCRPC, as per Quebec’s public healthcare system. The models include recently approved additional lines of treatment after or before docetaxel (i.e. abiraterone and cabazitaxel). Drug exposure and survival were based on clinical trial results and clinical practice guidelines found in a literature review. All costs were assigned in 2013 Canadian dollars ($). Only direct drug costs were estimated.

**Results:**

The mean cost of mCRPC drug treatments over an average period of 28.1 months was estimated at $48,428 per patient (95% Confidence Interval: $47,624 to $49,232). The mean cost increased to $104,071 (95% CI: $102,373 - $105,770) per patient when one includes abiraterone initiation prior to docetaxel therapy. Over the mCRPC period, luteinizing hormone-releasing hormone agonists (LHRHa) prescribed to maintain castrate testosterone levels accounted for 20.4% of the total medication cost, whereas denosumab prescribed to decrease bone-related events accounted for 30.5% of costs. When patients received cabazitaxel in sequence after abiraterone and docetaxel, the mCRPC medications cost per patient per month increased by 60.2%. The total cost of medications for the treatment of each annual Canadian cohort of 4,000 mCRPC patients was estimated at $ 193.6 million to $416.3 million.

**Conclusions:**

Our study estimates the direct drug costs associated with mCRPC treatments in the Canadian healthcare system. Recently identified effective yet not approved therapies will become part of the spectrum of mCRPC treatments, and may potentially increase the cost.

## Background

For Canadian men, prostate cancer (PCa) is the most common cancer and the 3rd leading cause of cancer mortality. As reported in 2012 by the Canadian Cancer Statistics, approximately 4,000 deaths were directly related to PCa [1].

It is well known that the growth of PCa is dependent on androgens. In the early 1940s, Huggins and Hodges demonstrated the importance of testosterone in PCa biology [2,3]. Since then, hormonal therapy (HT) or androgen ablation (ADT) has evolved from surgical to medical castration, initially achieved by luteinizing hormone-releasing hormone agonists (LHRHa) [4,5], and more recently, by gonadotropin-releasing hormone antagonists [6-11]. For patients with a high-risk of recurrence or progression of disease, medical and surgical castration remain the treatments of choice for hormone-sensitive PCa. Although surgical castration is much less expensive, the vast majority of patients are treated by medical castration.

ADT continues to play a key role in the treatment of advanced PCa. The 1st line of ADT for advanced PCa is an effective treatment that relieves symptoms (if present) and delays progression for several years [6,12]. However, virtually all of these patients will progress to a castrate resistant phase, known as castration-resistant PCa (CRPC) with a median survival of 30 months [13]. The start of the CRPC phase coincides with the rise in serum prostate-specific antigen (PSA) levels despite castrate levels of serum testosterone. Maintenance of ADT is believed to be important in CRPC*,* however the evidence remains weak [14,15].

The addition of an anti-androgen (AA) to block the effect of residual testosterone on the androgen receptor in patients medically or surgically castrated with ADT, helps to achieve maximum androgen blockade [16,17]. This is often considered as a 2nd line hormonal manipulation with a response rate of about 30% to 50% lasting for a mean duration of six months. Subsequently, anti-androgen withdrawal after relapse on maximum androgen blockade can result in an additional response rate of 20% to 30% for an average duration of four to five months [18,19]. Following anti-androgen treatment failure, further hormonal manipulation using adrenal androgen inhibitors may be considered [18]. Previously, ketoconazole was the agent of choice in this setting; however, ketaconazole use has decreased in the last few years because of its side effects, as well as emerging new evidence in favor of other hormonal treatments, such as abiraterone acetate [20]. Over a median follow-up period of 22.2 months, overall survival was superior in abiraterone-prednisone treated patients (median not reached) compared to patients receiving prednisone alone (median = 27.2 months). Furthermore, abiraterone showed superiority with respect to the time to initiation of cytotoxic chemotherapy (median time of 25.2 months in abiraterone-prednisone group and 16.8 months in prednisone-alone group).

During the CRPC period, patients often have distant metastases, with 90% of them bone-related [18,21]. This often causes severe pain as well as increases the risk of bone-related events such as pathologic fractures or spinal cord compression [22]. Therefore, supportive therapy targeting bone health using zoledronic acid or denosumab is indicated to decrease bone-related events [23-25].

Since 2004, cytotoxic chemotherapy with docetaxel has been the standard of care for metastatic CRPC (mCRPC) patients progressing on 1st- or 2nd- line ADT. Docetaxel showed significant yet modest improvements in survival (median of 3 months) and quality of life for patients with mCRPC [26,27]. Until recently, the therapeutic options for patients progressing on docetaxel were limited [28]. According to the most recent Canadian guidelines for the management of mCRPC [29,30], re-treatment with docetaxel can be considered for some patients [31,32]. Patients may also be treated with mitoxantrone. However, the spectrum of mCRPC treatment now includes several new treatment options, particularly for patients having already received docetaxel therapy. These treatments provide several additional months of survival compared to mitoxantrone [33]. Health Canada has recently approved three such novel drugs, cabazitaxel, abiraterone and enzalutamide [34-37]. Unfortunately, their high cost-effectiveness ratios have prompted provincial public healthcare systems in Canada to restrict access to public reimbursement. Consequently, in Quebec, access is totally restricted for cabazitaxel, whereas for abiraterone, access to the drug is only permitted for eligible mCRPC patients after docetaxel [38,39]. At the time of writing this manuscript, enzalutamide is not yet covered.

The contemporary management of mCRPC is very complex and is possibly associated with large drug costs. The main objective of this study was to develop a mathematical model to predict the total cost of medications associated with the most likely used mCRPC management strategies currently and in the near future, in the context of current evidence-based medicine treatment strategies applied to the Quebec healthcare system.

## Methods

This study was performed by using a modeling approach. Our modeling was based on Canadian clinical practice guidelines related to mCRPC and the results of clinical trials performed on this specific population. The selection of clinical trials was based on the target population, consisting of patients in the mCRPC phase receiving specific lines of treatment [17,18,20,26,34,36,40] as per Canadian clinical practice guidelines [29,30].

### Predictive model for the management of mCRPC

**Figure 1:**
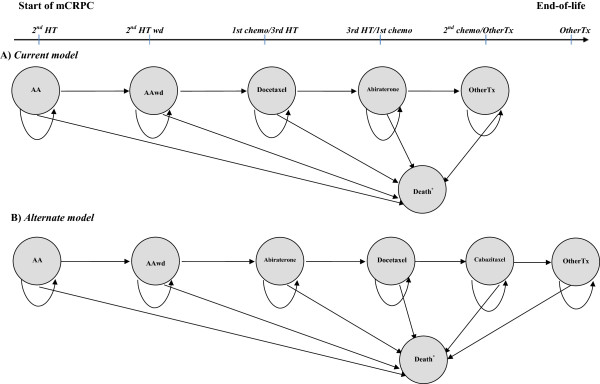
**Primary treatment sequence over mCRPC period: A) *Current model*; B) *Alternate model.*** Ovals/circles indicate treatment-related states. Straight arrows connecting two different treatment-related states show that the patient may move to a subsequent therapy state during each monthly cycle. Short curved arrows leading from a therapy state to itself indicate that the patient may remain in that state in successive cycles. *Abbreviations*: mCRPC = start of mCRPC state; 2nd HT = second line hormone therapy; 2nd HT wd = second line hormone therapy withdrawal; 1st chemo = first-line chemotherapy; 3rd HT = third line hormone therapy; 2nd chemo = second-line chemotherapy; OtherTx = Other treatments state; AA = anti-androgen state; AAwd = anti-androgen withdrawal state; *Death state integrates both prostate cancer related and non-related causes of death.

Two Markov models with Monte-Carlo microsimulations [41] were developed in order to simulate the management of the disease (treatment sequences) and to estimate the cost of drug treatments in mCRPC, as per Quebec’s public healthcare system and the latest drug developments. The model consists of distinct health states, which represent, clinically and economically important events that occur to patients in the mCRPC phase. All patients enter the first state. They may remain in that state (progression-free) in consecutive cycles, may move to either the subsequent state (progression) or can go to the dead state. The patients cannot return to the previous state. The health states were defined as treatment-related states since their sequence follows the treatment lines used in disease management. Because the mCRPC treatment pathway is related to the primary medication, the following general primary treatment sequence was assumed from the start of mCRPC: 1) 2nd HT, 2) 2nd HT withdrawal, 3) 1st chemotherapy/3rd HT, 4) 3rd HT/1st chemotherapy, 5) 2nd chemotherapy/Other treatments, and 6) Other treatments (OtherTx). Other treatments state can include chemotherapy re-treatment or other best supportive care therapies. Our first model was based on Canadian guidelines for the management of castrate-resistant PCa [29,30], using the list of medications approved for reimbursement by Quebec’s public insurance plan at the time of publication of these guidelines [42] (Figure 1A). This simulates the current treatment pathway and its associated cost of medications in Quebec in 2013. The following primary treatment sequence was assumed: 1) bicalutamide (AA); 2) bicalutamide withdrawal (AAwd); 3) docetaxel based-chemotherapy plus prednisone (docetaxel); 4) abiraterone acetate plus prednisone (abiraterone); and 5) OtherTx. This model reflects the most likely current management strategy of mCRPC in Quebec in 2013, and was named “*Current* model”. A second model was developed in the context of the latest evidence-based medicine for mCRPC management (Figure 1B) and was named “*Alternate* model”. The specific treatment sequence under the *Alternate* model was assumed as follows: 1) bicalutamide (AA); 2) bicalutamide withdrawal (AAwd); 3) abiraterone acetate plus prednisone (abiraterone); 4) docetaxel based-chemotherapy plus prednisone (docetaxel); 5) cabazitaxel based-chemotherapy plus prednisone (cabazitaxel); and 6) OtherTx.

The Markov model with Monte-Carlo microsimulations is a robust state-transition model that enables a dynamic treatment sequence to be simulated over the entire mCRPC period. The Monte Carlo microsimulation (also known as discrete event simulation) [43-45] is an individual level simulation that generates individual patient histories for outcomes (patient events and costs). Each simulated patient (trial) is included in the model and all patients’ events and costs will be accounted for during simulations by using tracker variables. Tracker variables add memory to the model and this allows us to know the simulated course for each patient. Essentially, it keeps track of the history of passage through the treatment-specific states during the simulation period as well as the associated costs. Our models simulate treatment sequences, duration of treatments and death in patients with mCRPC over one-month cycles. The models were built using TreeAge Pro 2013 (Release 13.1.1.0, TreeAge Software Inc.). The models simulate the patients’ transitions between treatment-related states until they die, or up to two years after they have completed the last available line of treatment. As patients with mCRPC often die of their cancer, no distinction was considered in our models for PCa related deaths. The mean cost per patient is the average of individual cost estimations obtained with the Monte-Carlo microsimulations. The 95% confidence interval (95% CI) for the mean cost was obtained through simulation of 1,000 samples of equal sample size.

### Transition probabilities

The transition probabilities among the different Markov models’ treatment-specific states were based on data obtained from selected studies [17,18,20,26,34,36]. Each line of treatment in mCRPC was documented in terms of treatment duration, progression-free survival and overall survival. The data are presented in Table 1. In order to obtain probabilities corresponding to one-month transition cycles, rates were converted using time-dependent monthly probabilities [41]. A minimum duration was assumed for each treatment in order to reach the median duration of treatment observed in clinical trials.

**Table 1:** Selected studies

**Primary medication**	**AA**	**AAwd**	**Abiraterone before docetaxel**	**Abiraterone after docetaxel**	**Docetaxel**	**Cabazitaxel**
**Source**	[17]	[18]	[20]	[34]	[26]	[36]
**Median duration of PSA response (months)**	12 (3–18)				7.7 (95% CI: 7.1-8.6)	
**Median time to PSA progression (months)**		5.9 (95% CI: 5.3-10.1)	11.1	10.2		6.4 (IQR: 2.2-10.1)
**Mean time to PSA progression (months)**						
**Progression free-survival (months)**			16.5	5.6		2.8 (95% CI: 2.4-3.0)
**Rate of mortality**	50%	50% at 16.7 months		42%	50% at 18.9 months	62%
	at the end of the study	&		at the time of preplanned interim analysis	&	at the time of cut-off
		90% at 60 months			82.8% at 3 years	
**Median survival time (months)**		16.7 (95% CI: 14.3 - 21.5)		14.8 (95% CI: 14.1-15.4)	18.9 (95% CI: 17–21.2)	15.1 (95% CI: 14.1-16.3)
**Median length of follow-up (months)**	24 (range: 3–54)		22.2	12.8	20.8	12.8 (IQR:, 7.8-16.9)
**Median cycles of treatment**				8 (range: 1–21)	9.5^*^ (range: 1–11)	6^*^ (IQR: 3–10)
**Median cycle of treatment (monthly equivalent)**					7.13 (range: 1–8.3)	5 (IQR: 2.3-7.5)

^*^3-weekly cycles.

*Abbreviations*: *AA* Anti-androgen, *AAwd* Anti-androgen withdrawal, *PSA* Prostate-specific antigen, *95% CI* 95% confidence interval, *IQR* Interquartile range.

### Cost assignments

All costs were assigned in 2013 Canadian dollars ($) and were estimated from the 2013 Quebec’s public healthcare system perspective. Therefore, the unit cost of each drug treatment was documented from the *Regie d’assurance maladie du Quebec* (RAMQ)’s list of medications approved for public reimbursement in Quebec [42]. Drug costs of chemotherapy administered in hospitals were based on the Montreal General Hospital pharmacy list [46] and a body surface area of 1.9 m^2^. This corresponds to the normal values reported for men in the general population [47]. Furthermore, as the sequence of treatment is not well defined in the OtherTx state (patients can participate to clinical trials or receive either docetaxel re-treatment, mitoxantrone or other best supportive therapies), the cost of primary treatments received was considered 0. Computation of costs was based on treatment protocols or regimens derived from clinical trials [17,18,20,26,34,36,40].

### Cost analyses

The cost of drug treatments in the mCRPC phase was estimated overall (total cost), by specific lines of treatment and categorized into: 1) the cost of primary drug treatment (drug acquisition, administration cost and pre-treatment medications used as prophylactic medications for chemotherapy-induced side effects e.g. dexamethasone); 2) the cost of medical castration for maintaining castrate testosterone levels (LHRHa); and 3) the cost of bone-targeted therapies, which consist of drugs used to prevent skeletal-related events (denosumab or zoledronic acid). Over the mCRPC period, 95% and 90% of patients were assumed to receive the medical castration and the bone-targeted therapy, respectively. The Canadian total drug cost of mCRPC was estimated by multiplying the number of patients that have journeyed to the mCRPC period before end-of-life (assumed to be equal to the number of PCa deaths in 2012 in Canada), by the mean cost of drugs over the mCRPC period.

### Sensitivity analysis

As there is no data available in the literature describing medication utilization rates in mCRPC (i.e. the proportion of patients progressing from one line of treatment to another), several scenarios were tested by varying (increasing and/or decreasing) the percentage of patients receiving the most expensive therapies. These scenarios were suspected to have the most important impact on the cost estimates. In addition, current population receiving specific treatment might be different from the population in clinical trials. As such, according to experts’ opinion, the following four scenarios have been considered plausible in actual clinical practice and have been tested. The *first scenario* assumed a 10% and 20% variation of the probability of transition from docetaxel to subsequent treatment. *Scenario 2)* assumed that 20%, 30% and 50% of patients received docetaxel re-treatment after docetaxel. *Scenario 3)* assumed that 50%, 70% and 100% of patients received maximum androgen blockade before entering the mCRPC phase, so their mCRPC phase starts at docetaxel (*Current* model) and abiraterone (*Alternate* model) initiation. Finally, *scenario 4)* assumed that 90%, 80% and 70% of patients received directly docetaxel (*Current* model) or abiraterone (*Alternate* model) after AA.

## Results

### Probabilities and cost estimations

The monthly probability of death, probability of transition to subsequent line of treatment and probability of staying in the same line of treatment were calculated for each line of treatment (data not shown). Unit and monthly costs of medications are listed in Table 2. Primary medication cost varied from $59 per patient per month for bicalutamide, to $8,460 per patient per month for cabazitaxel. The corresponding values for medical castration and bone-targeted therapy are estimated at $371 (cost of goserelin acetate) and $585 (cost of denosumab/zoledronic acid), respectively.

**Table 2:** Unit and monthly cost of mCRPC medications

**Medication**	**Dosage (reference)**		**Unit cost (reference)**		**Cost per month**
**Primary medication**					
Bicalutamide (Casodex)	1 × 50 mg daily	[17]	$1.61 per tablet (50 mg)	[42]	$58.71
Abiraterone (Zytiga)	4 × 250 mg daily	[34]	$28.3333 per tablet (250 mg)	[42]	$3,448
Cabazitaxel (Jevtana)	25 mg/m^2^ i.v. every 21 days	[36]	$5,840 per vial (60 mg/1.5 ml)	[46]	$8,460^*^
Docetaxel (Taxotere)	75 mg/m^2^ i.v. every 21 days	[26]	$599.79 per vial (160 mg/16 ml)^**^	[46]	$774^*^
Prednisone (Deltasone)	2 × 5 mg daily	[34]	$0.022 per tablet (5 mg)^**^	[42]	$1.34
**Premedication for chemotherapy-induced side effects**					
Dexamethasone^†‡^	8 mg i.v. every 21 days	[26,36]	$3.24 per vial (10 mg/ml, 1 ml)^**^	[46]	$4.69
Diphenhydramine^†^	25 mg i.v. every 21 days	[36]	$2.98 per vial (50 mg/ml, 1 ml)^**^	[46]	$4.32
Famotidine^†^	20 mg i.v. every 21 days	[36]	$2.71 per vial (10 mg/ml, 2 ml)^**^	[46]	$3.93
**Medication for medical castration**					
Goserelin (Zoladex)	10.8 mg s.c. every 3 months (13 weeks)	[40]	$1,113 per 10.8 mg depot 3 months	[42]	$371
**Bone-targeted therapy**					
Denosumab (XGEVA)	120 mg s.c. every 4 weeks	[23]	$538.45 per vial (120 mg/1.7 ml)	[42]	$585

^*^Based on a body surface area of 1.9 m^2^. This corresponds to the normal values reported for men in the general population [47]. The chemotherapies’ monthly cost was calculated under the assumption that the patients do not share the vials;

^**^Cost of generic.

^†^Premedication for cabazitaxel.

^‡^Premedication for docetaxel.

### Simulated treatment sequence, duration of treatments, survival in mCRPC and validity of the predictive models

Table 3 presents the percentage of patients who received each line of treatment, the duration of each treatment-specific state, and survival, as simulated by the *Current* and the *Alternate* models. Both models assumed that the mCRPC phase starts with anti-androgen blockade; therefore, all simulated patients start on the AA state. The *Current* model simulated that out of all patients starting with AA, 88.1% transited to AAwd, 72.5% received docetaxel, and 54.2% received abiraterone. With the *Alternate* model the corresponding values were: AAwd (88.0%), abiraterone (72.6%), docetaxel (47.4%), and cabazitaxel (34.4%). On follow-up, 29.4% and 26.9% of the patients, in the *Current* and the *Alternate* models, respectively, were still alive at the end of the treatment sequences and were transferred to OtherTx. Furthermore, the median duration of treatment in the *Current* model varied from 4 months (IQR: 3–6) for AAwd to 9 months (IQR: 4–17) for abiraterone, whereas it varied from 4 months (IQR: 3–6) for AAwd to 19 months (IQR: 9–35) for abiraterone in the *Alternate* model. The median overall survival in mCRPC was estimated at 25 months (IQR: 14–40) in the *Current* model, and 34 months (IQR: 15–56) in the *Alternate* model, respectively.

**Table 3:** Simulated treatment sequences, duration of treatments per patient, and survival in mCRPC

***Current model***						
**Treatment sequence**	**AA**	**AAwd**	**Docetaxel**	**Abiraterone**	**OtherTx**	
% of patients receiving each line of treatment	100%	88.1%	72.7%	54.2%	29.4%	
Mean duration per patient (95%CI)^*^	8.5 (8.4-8.6)	4.3 (4.3-4.4)	7.1 (7.0-7.1)	10 (9.9-10.2)	14.6 (14.3-14.9)	
Median duration per patient (IQR)^*^	8 (5–12)	4 (3–6)	7 (7–8)	9 (4–17)	15 (6–24)	
Mean Survival (95%CI)^**^	28.1 (27.7-28.4)	22.1 (21.7-22.4)	21.3 (20.9-21.6)	18.8 (18.4-19.1)	15.2 (14.9-15.5)	
Median Survival (IQR)^**^	25 (14–40)	19 (7–24)	18 (9–32)	16 (8–28)	16 (7–24)	
***Alternate model***						
**Treatment sequence**	**AA**	**AAwd**	**Abiraterone**	**Docetaxel**	**Cabazitaxel**	**OtherTx**
% of patients receiving each line of treatment	100%	88.0%	72.6%	47.4%	35.4%	26.9%
Mean duration per patient (95%CI)^*^	8.6 (8.5-8.7)	4.4 (4.3-4.4)	20.2 (19.9- 20.4)	7.0 (7.0-7.1)	5.7 (5.6-5.8)	15.0 (14.7-15.4)
Median duration per patient (IQR)^*^	8 (5–12)	4 (3–6)	19 (9–35)	7 (7–8)	5 (4–7)	16 (7–24)
Mean Survival (95%CI)^**^	37.3 (36.8-37.8)	32.5 (32.0-32.9)	33.8 (33.4.0-34.3)	20.4 (20.1-20.8)	17.6 (17.3-17.9)	15.7 (15.4-16.0)
Median Survival (IQR)^**^	34 (15–56)	30.0 (12–50)	32 (17–48)	18 (9–34)	17 (8–28)	17 (8–24)

^*^in each treatment-specific state (95% CI) (months); ^**^from entry into the treatment-specific state (IQR: 25 and 75 percentile);

*Abbreviations*: *95% CI* 95% confidence interval, *IQR* Interquartile range, *AA* Anti-androgen, *AAwd* Anti-androgen withdrawal, *OtherTx* Other treatments.

### Estimated cost of mCRPC medications by line of treatment

Table 4 presents the average cost of each line of treatment in patients receiving that line of treatment, the total and by type of medication. In the *Current* model among patients receiving docetaxel, the mean cost of docetaxel was estimated at $6,172 (95%CI: $6,126 - $6,219), the cost of medical castration while being in docetaxel state at $2,487 (95%CI: $2,467 - $2,506), and the cost of bone-targeted therapy at $3,715 (95%CI: $3,687 - $3,743), respectively, for a mean total cost of $12,374 (95%CI: $12,280 - $13,468). Moreover, among patients receiving abiraterone after docetaxel, the mean cost of primary medication was estimated at $34,624 (95%CI: $34,032 - $35,217), the cost of medical castration while being in abiraterone state at $3,538 (95%CI: $3,477 - $3,599), and the cost of bone-targeted therapy at $5,285 (95%CI: $5,195 - $5,376), respectively, for a mean total cost of $43,448 (95%CI: $42,706 - $44,192). In the *Alternate* model, among patients receiving abiraterone before docetaxel, the mean cost of abiraterone was estimated at $69,512 (95%CI: $68,593 - $70,431), the cost of medical castration while being in abiraterone state at $7,103 (95%CI: $7,009 - $7,197), and the cost of bone-targeted therapy at $10,611 (95%CI: $10,470 - $10,751), respectively, for a mean total cost of $87,227 (95%CI: $86,073 - $88,381). The corresponding values over the cabazitaxel sequence were: $49,131 (95%CI: $48,366 - $49,895), $2,007 (95%CI: $1,977 - $2,038), $2,999 (95%CI: $2,952 - $3,046), and $54,138 (95%CI: $53,295 - $54,980), respectively.

**Table 4:** Cost of primary medication, medication for medical castration, bone-targeted therapy per patient, by line of treatment of mCRPC

**Treatment sequence**	**Primary medication**		Medication for medical castration^*^		Bone-targeted therapy^**^		**Total**	
***Current model***	**Mean cost (95%CI)**	**Median cost (IQR)**	**Mean cost (95%CI)**	**Median cost (IQR)**	**Mean cost (95%CI)**	**Median cost (IQR)**	**Mean cost (95%CI)**	**Median cost (IQR)**
AA	$500	$410	$3,002	$2,467	$4,485	$3,686	$7,988	$6,563
	($495-$505)	($293-$705)	($2,972-$3,032)	($1,762-$4,229)	($4,440-$4,530)	($2,633-$6,318)	($7,907-$8,067)	($4,688-$11,251)
AAwd	$0	$0	$1,526	$1,409	$2,280	$2,106	$3,806	$3,515
			($1,513-$1,538)	($1,057-$2,114)	($2,261-$2,299)	($1,580-$3,159)	($3,775-$3,837)	($2,636-$5,273)
Docetaxel	$6,172	$6,124	$2,487	$2,467	$3,715	$3,686	$12,374	$12,277
	($6,126-$6,219)	($6,124-$6,999)	($2,467-$2,506)	($2,467-$2,819)	($3,687-$3,743)	($3,686-$4,212)	($12,280-$13,468)	($12,277-$14,031)
Abiraterone	$34,624	$31,040	$3,538	$3,172	$5,285	$4,739	$43,448	$38,950
	($34,032-$35,217)	($13,796-$58,631)	($3,477-$3,599)	($1,409-$5,991)	($5,195-$5,376)	($2,106-$8,950)	($42,706-$44,192)	($17,311-$73,573)
OtherTx	$0	$0	$5,130	$5,287	$7,664	$7,898	$12,794	$13,184
			($5,021-$5,239)	($2,115-$8,459)	($7,501-$7,862)	($3,159-$12,636)	($12,523-$13,065)	($5,274-$21,095)
***Alternate model***								
AA	$504	$470	$3,025	$2,819	$4,519	$4,212	$8,049	$7,501
	($498-$509)	($294-$704)	($2,995-$3,055)	($1,762-$4,229)	($4,474-$4,564)	($2,632-$6,318)	($7,968-$8,128)	($4,688-$11,251)
AAwd	$0	$0	$1,541	$1,410	$2,303	$2,106	$3,844	$3,515
			($1,529-$1,554)	($1,057-$2,115)	($2,283-$2,321)	($1,580-$3,159)	($3,812-$3,874)	($2,637-$5,273)
Abiraterone	$69,512	$65,531	$7,103	$6,697	$10,611	$10,003	$87,227	$82,231
	($68,593-$70,431)	($31,041-$120,715)	($7,009-$7,197)	($3,172-$12,335)	($10,470-$10,751)	($4,738-$18,428)	($86,073-$88,381)	($38,952-$151,478)
Docetaxel	$6,143	$6,118	$2,477	$2,467	$3,701	$3,686	$12,321	$12,271
	($6,083-$6,203)	($6,118-$6,992)	($2,453-$2,501)	($2,467-$2,819)	($3,665-$3,737)	($3,686-$4,212)	($12,201-$12,441)	($12,271-$14,024)
Cabazitaxel	$49,131	$43,125	$2,007	$1,762	$2,999	$2,632	$54,138	$47,519
	($48,366-$49,895)	($34,500-$60,375)	($1,977-$2,038)	($1,409-$2,467)	($2,952-$3,046)	($2,106-$3,685)	($53,295-$54,980)	($38,016-$66,528)
OtherTx	$0	$0	$5,300	$5,639	$7,918	$8,424	$13,217	$14,063
			($5,186-$5,414)	($2,467-$8,459)	($7,747-$8,087)	($3,685-12,636)	($12,933-$13,501)	($6,153-$21,095)

*Abbreviations*: *95%CI* 95% confidence interval, *IQR* Interquartile range, *AA* Anti-androgen, *AAwd* Anti-androgen withdrawal, *OtherTx* Other treatments.

^*^assuming that only 95% of patients have received medication for medical castration; ^**^assuming that only 90% of patients have received bone-targeted therapy.

### Total cost of medications over the mCRPC period

Under the *Current* model, the total cost of medications over the entire mCRPC period was estimated at $48,428 (95%CI: $47,624 - $49,232) per patient (Table 5). Primary medications (AA, docetaxel and abiraterone) accounted for 49.1% of the total cost, medical castration for 20.4%, and bone-targeted medications for 30.5%. The corresponding value in the *Alternate* model was: $104,071 (95%CI: $102,373 - $105,770), from which primary medications accounted for 68.5%, medical castration for 12.6% and bone-targeted medication for 18.9%. Furthermore, the monthly cost of medications in mCRPC increased by 61.9% in the *Alternate* model*.* The cost for medical castration and bone-targeted medication increased by 32.8% (due to the difference in mCRPC duration of 28.1 months in the *Current* model and 37.3 months in the *Alternate* model), whereas the cost of primary medication tripled compared to the *Current* model. This increase is attributed to the cost of cabazitaxel (35.4%), doubled treatment duration of abiraterone (38.5%) (median of 9 months when administered after docetaxel, compared to 19 months when administered before docetaxel), as well as 18.4% more patients receiving abiraterone when prescribed before docetaxel (26.1%).

**Table 5:** Total and monthly drugs cost of mCRPC by type of medication

	**Total mCRPC cost per patient**		**Monthly mCRPC cost per patient**	**Total mCRPC cost in Canada**	
***Current model***	**Mean (95%CI)**	**Median (IQR)**	**Mean (95%CI)**	**(annual cohort of 4,000 mCRPC patients)**	
**Medication type:**					
Primary medication^*^	$23,745 ($23,238-$24,253)	$13,316 ($939-$41,553)	$845 ($827-$882)	$94,980,000	49.1%
Medication for medical castration^†^	$9,898 ($9,779-$10,016)	$8,811 ($4,934-$14,098)	$352 ($348-$357)	$39,592,000	20.4%
Bone-targeted therapy^‡^	$14,785 ($14,607-$14,963)	$13,163 ($7,371-$21,060)	$526 ($520-$532)	$59,032,000	30.5%
**Total cost of mCRPC**	$48,428 ($47,624-$49,232)	$35,290 ($13,244-$76,711)	$1,723 ($1,695-$1,752)	$193,604,000	100.0%
***Alternate model***					
**Medication type:**					
Primary medication^**^	$71,302 ($70,026-$72,579)	$62,816 ($939-$123,501)	$1,912 ($1,877-$1,946)	$285,208,000	68.5%
Medication for medical castration^†^	$13,140 ($12,971-$13,309)	$11,983 ($5,639-$19,737)	$352 ($348-$357)	$52,560,000	12.6%
Bone-targeted therapy^‡^	$19,629 ($19,376-$19,882)	$17,901 ($8,424-$29,484)	$526 ($520-$532)	$78,516,000	18.9%
**Total cost of mCRPC**	$104,071 ($102,373-$105,770)	$92,700 ($15,002-$172,722)	$2,790 ($2,745-$2,835)	$416,284,000	100.0%

*Abbreviations*: *95%CI* 95% confidence interval, *IQR* Interquartile range;

^*^includes AA, docetaxel and abitarerone;

^**^includes AA, abitarerone, docetaxel and cabazitaxel;

^†^assuming that only 95% of patients have received medication for medical castration;

^‡^assuming that only 90% of patients have received bone-targeted therapy.

**Figure 2:**
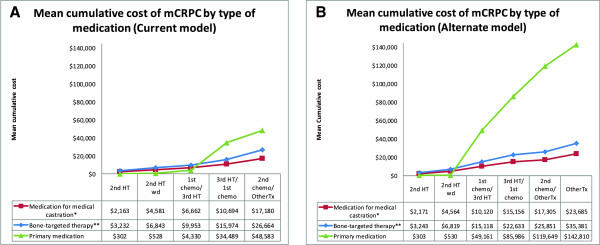
**Mean cumulative drugs cost per patient over the mCRPC treatment sequences, by type of medication; A) *Current *model and B) *Alternate *model. ***Abbreviations*: 2nd HT = second line hormone therapy; 2nd HT wd = second line hormone therapy withdrawal; 1st chemo = first-line chemotherapy; 3rd HT = third line hormone therapy; 2nd chemo = second-line chemotherapy; OtherTx = Other treatments state; ^*^assuming that only 95% of patients have received medication for medical castration; ^**^assuming that only 90% of patients have received bone-targeted therapy.

Figure 2 shows that depending on the last line of treatment received in the *Current* model, the total cost of mCRPC varied from $5,697 per patient (in group receiving only AA; $2,163 for medical castration medication, $3,232 for bone-targeted therapy, and $302 for primary medication) to $92,427 per patient (in group receiving all lines of treatment; $17,180 for medical castration medication, $26,664 for bone-targeted therapy, and $48,583 for primary medication). The corresponding values in the *Alternate* model were from $5,717 to $201,875.

### Sensitivity analysis

The results of the sensitivity analysis are presented in Table 6. Various scenarios were formulated and the results were consistent with the primary results; except for the scenarios when the entry into the mCRPC phase corresponded with docetaxel (*Current model*) and abiraterone (*Alternate model*) initiation for 50%, 70% and 100% of patients, respectively. In these scenarios, the mean cost per patient per month increased up to $500 in the *Current model* and up to $1,000 in the *Alternate model*.

**Table 6:** Sensitivity analysis

	**mCRPC cost (per patient)**		**Monthly mCRPC cost**
	**Mean (95%CI)**	**Median (IQR)**	**Mean (95%CI)**
**Scenario 1: Variation of the probability of transition from docetaxel to subsequent treatment**			
*Current model*			
*10*% *increase of transition to abiraterone*	$48,049 ($47,451-$48,778)	$39,395 ($14,465-$77,508)	$1,743 ($1,721-$1,769)
*20*% *increase of transition to abiraterone*	$48,096 ($47,467-$48,817)	$39,669 ($13,888-$76,884)	$1,766 ($1,743-$1,793)
*10*% *decrease of transition to abiraterone*	$48,026 ($47,253-$48,697)	$36,064 ($14,006-$75,805)	$1,707 ($1,680-$1,731)
*20*% *decrease of transition to abiraterone*	$48,049 ($47,125-$48,783)	$36,402 ($14,407-$76,710)	$1,706 ($1,673-$1,732)
*Alternate model*			
*10*% *increase of transition to cabazitaxel*	$102,998 ($101,424-$104,765)	$92,113 ($16,165-$170,454)	$2,814 ($2,771-$2,862)
*20*% *increase of transition to cabazitaxel*	$102,846 ($101,065-$104,960)	$90,435 ($16,760-$171,956)	$2,833 ($2,784-$2,891)
*10*% *decrease of transition to cabazitaxel*	$103,626 ($102,001-$105,506)	$94,951 ($16,466-$171,206)	$2,801 ($2,757-$2,852)
*20*% *decrease of transition to cabazitaxel*	$103,784 ($102,169-$105,513)	$95,900 ($16,165-$173,512)	$2,802 ($2,758-$2,849)
**Scenario 2: 20%, 30% and 50% of patients received docetaxel retreatment after docetaxel**			
*Current model*			
*20*% *of patients received docetaxel retreatment*	$45,312 ($44,693-$45,873)	$34,732 ($14,469-$71,373)	$1,615 ($1,593-$1,635)
*30*% *of patients received docetaxel retreatment*	$43,925 ($43,302-$44,522)	$34,592 ($13,888-$68,665)	$1,565 ($1,543-$1,587)
*50*% *of patients received docetaxel retreatment*	$41,182 ($40,607-$41,964)	$32,188 ($13,649-$62,930)	$1,467 ($1,447-$1,495)
*Alternate model*			
*20*% *of patients received docetaxel retreatment*	$99,887 ($98,628-$101,220)	$90,204 ($15,764-$168,696)	$2,705 ($2,671-$2,741)
*30*% *of patients received docetaxel retreatment*	$98,335 ($96,877-$99,735)	$88,783 ($16,643-$164,680)	$2,660 ($2,620-$2,698)
*50*% *of patients received docetaxel retreatment*	$95,227 ($93,735-$96,347)	$88,644 ($16,584-$163,683)	$2,577 ($2,537-$2,607)
**Scenario 3: 50%, 70% and 100% received AA before mCRPC phase**			
Current model^*^			
*50*% *of patients received AA before entering mCRPC phase*	$48,378 ($47,841-$49,042)	$39,452 ($13,829-$76,165)	$2,160 ($2,136-$2,189)
*70*% *of patients received AA before entering mCRPC phase*	$48,459 ($47,750-$49,169)	$39,452 ($14,006-$77,391)	$2,233 ($2,200-$2,266)
*100*% *of patients received AA before entering mCRPC phase*	$48,532 ($47,780-$49,329)	$40,876 ($13,155-$75,612)	$2,357 ($2,321-$2,396)
Alternate model^**^			
*50*% *of patients received AA before entering mCRPC phase*	$113,645 ($112,019-$115,416)	$109,405 ($37,800-$175,884)	$3,433 ($3,384-$3,487)
*70*% *of patients received AA before entering mCRPC phase*	$117,895 ($116,248-$119,549)	$112,648 ($44,486-$174,101)	$3,562 ($3,512-$3,612)
*100*% *of patients received AA before entering mCRPC phase*	$124,300 ($123,133-$125,472)	$122,061 ($59,878-$177,657)	$3,758 ($3,722-$3,793)
**Scenario 4: Variation of the rate of patients transiting to docetaxel (Current model)/abiraterone (Alternate model)**^**†**^			
*Current model*			
*90*% *of patients transit to docetaxel*	$51,936 ($51,147-$52,584)	$44,070 ($17,843-$80,975)	$1,893 ($1,865-$1,917)
*80*% *of patients transit to docetaxel*	$51,571 ($50,769-$52,390)	$43,459 ($16,760-$80,710)	$1,873 ($1,844-$1,903)
*70*% *of patients transit to docetaxel*	$51,106 ($50,399-$51,797)	$42,377 ($16,333-$79,588)	$1,851 ($1,825-$1,876)
*Alternate model*			
*90*% *of patients transit to abiraterone*	$117,515 ($115,852-$118,932)	$115,641 ($45,674-$177,184)	$3,086 ($3,042-$3,123)
*80*% *of patients transit to abiraterone*	$115,974 ($114,204-$117,959)	$113,188 ($42,052-$176,342)	$3,054 ($3,008-$3,107)
*70*% *of patients transit to abiraterone*	$114,245 ($112,716-$115,980)	$111,760 ($37,922-$176,258)	$3,022 ($2,981-$3,067)

^*^mCRPC starts at docetaxel initiation for 50%, 70% and 100% of patients;

^**^mCRPC starts at abiraterone initiation for 50%, 70% and 100% of patients;

^†^After AA: 10%, 20% and 30% of patients transit to AAwd, and respectively, 90%, 80% and 70% of patients transit to Docetaxel (*Current* model) and Abiraterone (*Alternate* model).

*Abbreviations*: *95%CI* 95% confidence interval, *IQR* Interquartile range.

## Discussion

Our study highlights the important economic burden related to medications over the entire period of mCRPC, based on current management of the disease in Quebec and the latest drug developments. In Canada, over a mean period of 28.1 months (estimated with the *Current* model) the total cost of mCRPC medications associated with the most likely current management strategies of an annual cohort of 4,000 patients was estimated at $193.6 million. For an equivalent period of time, if abiraterone was offered to patients before docetaxel and cabazitaxel, the total cost was increased to $313.5 million, and up to $416.3 million over a mCRPC mean duration of 37.3 months (estimated with the *Alternate* model).

Our models simulated durations of treatment that were very similar to those reported in clinical trials. However, since the median duration of abiraterone before docetaxel was not reported, the median duration of abiraterone of 19 months (obtained with the *Alternate* model), was compared to the median time to radiographic progression-free survival of 16.5 months, and to median time to cytotoxic chemotherapy initiation of 25 months, as reported in the clinical trial [20].

Some differences were also observed in survival from initiation of a particular line of treatment, compared to survival showed in clinical trials. This is mainly explained by the fact that in clinical trials, treatments are evaluated individually across a line of treatment, and not in sequence with a preceding or subsequent line of treatment. However, the median overall survival estimated from the start of mCRPC was comparable to that reported in the literature [13]. Furthermore, a 9 month difference in mean duration of mCRPC was observed between the *Alternate* model and the *Current* model (37.3 versus 28.1 months). We believe this can be explained by the difference in disease severity between patients that received abiraterone before docetaxel (asymptomatic mCRPC) compared with patients that received abiraterone after docetaxel (symptomatic mCRPC). Further evidence is required to better understand if the financial effort of offering abiraterone before docetaxel is translated into a real clinical advantage over the strategy of offering abiraterone after docetaxel. The *Alternate* model showed that 65% of the increase of mCRPC cost is attributable to this sequence change. It is important to understand if this cost difference is due to overtreatment or not of asymptomatic patients. Besides the fact that our study was not designed to respond to this question, our results highlight the financial implication of this management strategy.

Provincial or federal authorities, such as the *Institut national d’excellence en santé et en services sociaux* (INESSS) and the pan-Canadian Oncology Drugs Review (pCODR), assess new drugs in the treatment of mCRPC or other cancers through an evaluation process based primarily on the results of clinical trials and cost estimates. Each new drug is evaluated individually across a particular line of treatment, with limited consideration to the public reimbursement impact of the global cost of mCRPC. To the best of our knowledge, there is no study specific to the Canadian context or elsewhere, which evaluates the total cost of mCRPC medications in the changing landscape of mCRPC management. On the other hand, there are a few studies evaluating the direct costs associated with end-of-life care in PCa patients, particularly within the last 6 to 18 months of life [48-50]. However, these reported costs did not include the costs of newer treatments, such as abiraterone, cabazitaxel, and denosumab. In the pre-docetaxel era, the cost of prostate cancer-specific drugs over the last 18 months of life was estimated at $1,695 per patient (2004 Canadian $) [48]. In addition, two studies reported the cost of drugs in both the pre- and post- docetaxel eras [50]. A mean cost of US$72 was estimated among a cohort with less than 25% of patients having received docetaxel. Furthermore, the mean cost of drugs per-month-per-patient rose more than 6 times, from US$254 in the pre-docetaxel period to US$1,623 in the post-docetaxel period [51]. These figures included drugs such as docetaxel, other chemotherapies (mitoxantrone), androgen deprivation therapies, ketoconazole and prednisone.

This study has several limitations worth mentioning. The predictive models were based on treatment sequences and the probabilities of transition were derived from clinical trials. This leads to two main concerns. Firstly, the characteristics of patients participating in clinical trials are often different from those of the simulated population, and thus, the results cannot be generalized. For example, there is no study evaluating cabazitaxel on patients having previously received abiraterone before or after docetaxel, yet cabazitaxel is likely to be used in sequence after docetaxel and abiraterone. Secondly, our estimated cost is a predicted cost of medications in mCRPC, rather than an actual cost. Furthermore, the estimates do not include the cost of other primary medications that patients can receive after abiraterone (*Current* model) or cabazitaxel (*Alternate* model), such as mitoxantrone or docetaxel re-treatment. In this context, a cohort study is suitable to estimate the actual treatment pathway and the actual cost of mCRPC based on real-life data and to capture the true variability of treatment choices. Our study is restricted to the most likely treatment sequences in Quebec in 2013. However, by conducting the sensitivity analysis, we measured the impact of the most important assumptions of the models on the cost estimates. Another limitation is that this cost evaluation was based mainly on costs derived from Quebec’s RAMQ. Nevertheless, the cost of medications is generally similar across Canadian provinces. Consequently, the transferability of these results is reasonable to Canada. Third, the cost of adverse events associated with primary therapy (i.e. febrile neutropenia, stomatitis and diarrhea) was not considered; therefore the cost of mCRPC could be underestimated. In addition, our cost estimates are only drug-related. The total cost of mCRPC that includes palliative radiotherapy would be much higher. In addition, the impact of the very recent approval of enzalutamide for mCRPC failing docetaxel chemotherapy will have to be evaluated once provincial authorities provide reimbursement. Finally, the principal limitation of our study, as well as others employing modeling, is the reliance on estimates as opposed to prospective cohort evaluations. However, the newer treatment options (abiraterone and cabazitaxel) were not available before 2012, and consequently, our modeling approach is the only possible alternative to estimate cost at the present time and in the near future. Reassuringly, the simulated durations for each treatment were similar to those reported in clinical trials, which confirm the validity of our model and, correspondingly the accuracy of our cost estimates. Finally, our study does not compare the cost-effectiveness ratios associated with these treatments since it is obvious that these approved drugs deliver clinical benefits including prolonged survival.

Other encouraging developments in mCRPC treatment have recently been revealed [52]. These include drugs such as TAK-700 (orteronel), a non-steroidal, selective inhibitor of the 17,20-lyase activity of CYP17A--a key enzyme in the production of steroidal hormones [52,53], as well as radium-223 dichloride (radium-223), a targeted alpha emitter that selectively binds to areas of increased bone turnover in bone metastases [54]. In addition, the US Food and Drug Administration has approved an immune based therapy, called sipuleucel-T for patients with metastatic disease and minimal symptoms [55]. Other therapies primarily related to anti-angiogenesis [56] could potentially be part of the spectrum of mCRPC treatment. Thus, it is possible that after a short period of time, the management of mCRPC will become even more complex and the associated drugs cost even higher.

It is clear that there is a lack of knowledge examining the contemporary cost of PCa management, particularly in the advanced stages. Our study helps demonstrate the costs associated with various specific mCRPC management strategies. We hope that such information will support decision makers in their process of evaluation of new drugs by integrating the economic evaluation of the overall disease management in addition to the traditional sequential line of treatment approach. Furthermore, this will help clinicians be more aware of the financial impact of their medical decisions. There is no doubt that in order to afford new expensive treatments, one must find cost savings elsewhere in the disease management process. For example, continuous medical castration during the mCRPC stage is associated with significant costs, yet questionable benefit. The costs of new and expensive therapies for mCRPC can perhaps partially be recovered by the decreased use of LHRHa therapies, estimated to account for 21% of the total cost of mCRPC.

## Conclusions

In conclusion, over a mean period of less than three years, we showed that the cost of mCRPC medications is significant and susceptible to increase in the near future to prohibitive levels. In the current setting of rising treatment and drug costs, especially for the treatment of advanced cancers, the economic burden on the Canadian healthcare system and Canadians has increased dramatically. Therefore, access to cancer drugs for mCRPC in the Canadian healthcare system has become challenging. With our model, we are able to simulate the management of mCRPC and its associated costs over this period. This can be a valuable tool for decision makers and clinician leaders, helping influence decisions about public access to innovative treatments, and assisting in achieving optimal management for these patients. Furthermore, this model is able to provide a real-time estimation of the financial impact on the total cost of mCRPC of a certain decision at a particular level over the course of mCRPC treatment.

## Abbreviations

PCa: Prostate cancer; mCRPC: Metastatic castration-resistant prostate cancer; HT: Hormonal therapy; LHRHa: Luteinizing hormone-releasing hormone agonists; PSA: Prostate-specific antigen; CRPC: Castration-resistant prostate cancer; ADT: Androgen deprivation therapy/androgen ablation; AA: Anti-androgen; AAwd: Anti-androgen withdrawal; OtherTx: Other treatments; 95%CI: 95% confidence interval; IQR: Interquartile range, INESSS*, Institut national d’excellence en santé et en services sociaux*; pCODR: Pan-Canadian Oncology Drug Review.

## Competing interests

The authors declare that they have no competing interests.

## Authors’ contributions

AD and AGA designed the study. AD, DD, MV, FLC and AGA participated in model development and in cost assignments. AD, DD, MV, FLC and AGA participated in selection of clinical trials and health states sequence used for model development. AD conducted the statistical analysis and all authors participated to interpretation of the results. AD and AGA participated in drafting the manuscript. All authors provided critical commentary on the manuscript and approved the final version.

## Pre-publication history

The pre-publication history for this paper can be accessed here:

http://www.biomedcentral.com/1472-6963/14/252/prepub
